# Clinical stage 1 non-Hodgkin's lymphoma: long-term follow-up of patients treated by the British National Lymphoma Investigation with radiotherapy alone as initial therapy.

**DOI:** 10.1038/bjc.1994.213

**Published:** 1994-06

**Authors:** B. Vaughan Hudson, G. Vaughan Hudson, K. A. MacLennan, L. Anderson, D. C. Linch

**Affiliations:** Department of Haematology, University College London School of Medicine, UK.

## Abstract

A retrospective analysis was performed of 451 adult patients with clinical stage 1/1E non-Hodgkin's lymphoma treated initially with radiotherapy alone. Histopathologically 208 patients had low-grade disease and 243 patients high-grade disease. The complete remission (CR) rate was higher in patients with low-grade disease (98%) than in those with high-grade disease (84%) (P < 0.0001). The relapse rate was similar in both histological categories, and relapse usually occurred within 5 years. The resulting overall actuarial percentage of patients achieving CR and remaining disease free (at 10 years) was 47% in patients with low-grade disease and 45% for those with high-grade disease. Salvage therapy was frequently successful in younger patients, and the overall cause-specific survival at 10 years was 71% for low-grade disease and 67% for high-grade disease. In those patients under 60 years of age at diagnosis, the overall cause-specific survival at 10 years was 84% and 80% for those with low-grade and high-grade disease respectively. These long-term results in young patients with clinical stage 1 disease are encouraging, and it will be difficult to demonstrate improved survival with initial chemotherapy either with or without radiotherapy, until new prognostic factors are found to identify poor-risk patients.


Br. J. Cancer (1994), 69, 1088-1093 © Macmillan Press Ltd., 1994

### Clinical stage 1 non-Hodgkin's lymphoma: long-term follow-up of patients treated by the British National Lymphoma Investigation with radiotherapy alone as initial therapy

B. Vaughan Hudson[1], G. Vaughan Hudson[1], K.A. MacLennan[2], L. Anderson[1] & D.C. Linch[1]

[1]*The British National Lymphoma Investigation, Departments of Haematology and Oncology, University College London School of Medicine, London W1N 8AA, UK;* [2]*Institute for Cancer Studies, St James' University Hospital, Leeds LS9 7TF, UK.*

**Summary** A retrospective analysis was performed of 451 adult patients with clinical stage 1/1E non-Hodgkin's lymphoma treated initially with radiotherapy alone. Histopathologically 208 patients had low-grade disease and 243 patients high-grade disease. The complete remission (CR) rate was higher in patients with low-grade disease (98%) than in those with high-grade disease (84%) ($P<0.0001$). The relapse rate was similar in both histological categories, and relapse usually occurred within 5 years. The resulting overall actuarial percentage of patients achieving CR and remaining disease free (at 10 years) was 47% in patients with low-grade disease and 45% for those with high-grade disease. Salvage therapy was frequently successful in younger patients, and the overall cause-specific survival at 10 years was 71% for low-grade disease and 67% for high-grade disease. In those patients under 60 years of age at diagnosis, the overall cause-specific survival at 10 years was 84% and 80% for those with low-grade and high-grade disease respectively. These long-term results in young patients with clinical stage 1 disease are encouraging, and it will be difficult to demonstrate improved survival with initial chemotherapy either with or without radiotherapy, until new prognostic factors are found to identify poor-risk patients.

Disease apparently localised to a single nodal group at presentation (stages 1 and 1E) is not infrequent in the non-Hodgkin's lymphomas. In the series of 3,924 clinically staged patients with NHL entered into trials and studies of the British National Lymphoma Investigation (BNLI), 824 (21%) patients have stage 1 or 1E disease. Laparotomy was rarely carried out as a staging procedure for these patients but would have resulted in a lower incidence of stage 1 and 1E disease.

Optimal management of NHL depends on knowledge not only of the anatomical stage, but also of the histological grade. It is usual practice to treat those stage 1/1E patients who have histologically indolent (low-grade) lymphomas with radiotherapy (RT) (O'Reilly & Connors, 1992), and there is no evidence that additional chemotherapy (CT) improves the outlook (Nissen *et al.*, 1983). In pathological stage 1 and 1E patients with histologically aggressive (high-grade) lymphomas radiotherapy is curative in approximately 90% of patients (Miller & Jones, 1980; Vokes *et al.*, 1985; Hallahan *et al.*, 1989), providing that those rare cases of localised lymphoblastic and small non-cleaved cell lymphomas which have a high risk of dissemination (particularly to the CNS) are excluded. Few centres would consider a staging laparotomy to be justified in non-Hodgkin's lymphoma, and in clinically staged patients the relapse rate and subsequent mortality have been reported to be considerably higher than in laparotomised patients (Hallahan *et al.*, 1989), thus leading to the widespread use of combination chemotherapy with or without radiotherapy. These initial studies were, however, small and must be interpreted with caution. Numerous subsequent series of patients have been reported, but many of these have also been small, or have combined stage 1 and stage II patients, laparotomised and non-laparotomised patients, both low- and high-grade histology, or both initial RT and CT, thus making detailed interpretation difficult. A retrospective analysis was therefore carried out upon those patients with clinical stage 1/1E NHL treated with initial radiotherapy alone registered on the BNLI database. Patients with low- and high-grade lymphomas were analysed separately and the results compared. Patients with lymphoblastic, small non-cleaved cell lymphomas or with testicular lymphomas are not included in this series as they have a high risk of dissemination to the CNS and probably require CNS prophylaxis (Nissen & Ersboll, 1985; Crellin *et al.*, 1993). Primary gut lymphomas have also been excluded as many of these appear to be a distinct biological entity (Isaacson & Wright, 1984; Isaacson & Spencer, 1988) and we have reported our results for these tumours elsewhere (Morton *et al.*, 1993).

Correspondence: G. Vaughan Hudson, BNLI, Department of Oncology, The Middlesex Hospital, Mortimer Street, London W1N 8AA, UK.
Received 21 October 1993; and in revised form 20 January 1994

### Patients and methods

### *Patients*

A retrospective analysis has been made of 451 adult patients (age>16 years) with clinical stage 1/1E disease without B symptoms, entered into BNLI trials and studies during the period 1974-91, for whom radiotherapy alone was the planned initial therapy. Patients with lymphoblastic, non-cleaved cell lymphomas, testicular lymphomas or gut lymphomas were excluded from the analysis. All patients fulfilling the entry criteria were included, although we acknowledge that in a multicentre study it is not possible to fully exclude an element of selection bias in the entry. No patients had a staging laparotomy, and the means of seeking intra-abdominal disease changed over the time span of this series. In the early 1970s most patients had lymphangiograms and isotopic liver scans, whereas in the 1980s these were largely replaced by computerised tomography. All patients in this series had a staging bone marrow biopsy which was negative. Precise measurements of tumour masses at diagnosis were not recorded in the early stages of this series and so it was not possible to accurately identify patients with bulky disease.

The histopathology was assessed in all cases by the central BNLI pathology panel. Low-grade disease is defined here as follicular lymphomas, diffuse lymphocytic lymphoma and diffuse small cleaved lymphoma. High-grade disease refers to diffuse mixed small- and large-cell lymphoma, diffuse large-cell lymphoma and diffuse immunoblastic lymphoma.

In all the treatment protocols over the period of patient entry, a dose of 3,500 cGy was suggested for low-grade lymphomas and a minimum dose of 4,000 cGy for high-grade lymphomas. Details of the doses given and the radiation fields are not available for the majority of patients.

*Statistical analysis*

Overall survival included deaths from all causes, while cause-specific survival deaths which were not due to NHL or its treatment were censored. Survival was calculated by the life-table method, and statistical comparison of curves made by means of the log-rank test as described by Peto *et al.* (1971). Multivariate analysis was performed by the use of a stepwise proportional hazards model due to Cox (1972), the variables included in the analysis being age, sex, type (nodal or extra-nodal) and site of involvement, histopathological subtype and grade and presentation lymphocyte count. Nodal involvement was subdivided into cervical, axillary, groin and other sites. Extranodal involvement was arbitrarily divided on the basis of frequency of occurrence into six groups, consisting of those sites of involvement occurring with a frequency of occurrence of >10 (which comprised tonsil, skin, thyroid, parotid and eye/orbit), and those occurring with a frequency of 10 or less.

### Results

*Low-grade disease*

*Patient demographics* Two hundred and eight patients had low-grade stage 1 disease, which was nodal in 149 cases (72%) and extranodal in 59 cases (28%). Of these patients, 81 had follicular small-cell lymphoma, 72 follicular mixed-cell lymphoma, ten follicular large-cell lymphoma, 27 diffuse lymphocytic lymphoma and 18 diffuse small cleaved cell lymphoma. There were 110 males and 98 females. The median age was 59 years with a range of 31–86 years. The sites of disease are shown in Table I.

Nine patients had a Hb<12 g $dl^{-1}$ at presentation, six had an albumin of <36 g $l^{-1}$ and seven patients had an erythrocyte sedimentation rate (ESR) of 40 mm $h^{-1}$ or more. Fifty-seven patients (27%) had a low lymphocyte count (<1.5 × $10^9\ l^{-1}$) at presentation.

*Results of treatment* Two hundred and three patients (98%) achieved a complete remission (CR) maintained for at least 3 months after completion of therapy. Of the five patients who did not achieve a CR, two achieved a CR with subsequent chemotherapy. Of the patients achieving a CR with initial RT the actuarial relapse rate at 10 years was 51%, most of the relapses occurring within 5 years with few thereafter.

The resulting overall actuarial percentage of patients achieving CR and remaining disease free thereafter was 47% at 10 years.

Of the 72 patients who received second-line treatment (five after induction failure and 67 on relapse), 34 (47%) attained a second CR from it (after radiotherapy in 17, and after chemotherapy – usually chlorambucil – in 17).

The overall actuarial survival at 10 years was 64%, rising to 71% when patients dying from causes other than lymphoma or its treatment were excluded.

*Subgroup analysis* Multivariate analysis of the relapse rate of those patients with low-grade disease who achieved complete remission from their initial treatment identified age as the only significant factor related to relapse rate ($P<0.04$), with older patients having a higher relapse rate than younger ones. The CR rates of patients aged <50, 50–59, 60–69 and 70+ were similar at 100%, 96%, 96% and 100% respectively. The percentage of patients of different ages remaining disease free from their initial treatment alone is shown in Figure 1.

Multivariate analysis revealed age to be the only significant factor related to survival ($P<0.0007$), with older age being related to relatively poor survival. The survival of patients in the different age groups is shown in Figure 2. Of patients

**Table I** Stage 1/1E NHL: sites of involvement

| | *Low grade* | *High grade* |
|---|---|---|
| *Nodal* | | |
| Neck | 65 (31%) | 82 (34%) |
| Axilla | 15 (7%) | 19 (8%) |
| Inguinal | 67 (32%) | 42 (17%) |
| Other | 2 (1%) | 2 (1%) |
| Total | 149 (72%) | 145 (60%) |
| *Extranodal* | | |
| Tonsil | 8 | 24 |
| Skin | 8 | 19 |
| Thyroid | 7 | 16 |
| Parotid | 16 | 6 |
| Eye/orbit | 10 | 2 |
| Nasopharynx | 0 | 7 |
| Bone | 0 | 6 |
| Brain | 0 | 5 |
| Tongue | 2 | 3 |
| Palate | 1 | 3 |
| Extradural | 1 | 2 |
| Larynx | 1 | 1 |
| Breast | 2 | 0 |
| Gum | 1 | 1 |
| Thymus | 0 | 1 |
| Cervix | 0 | 2 |
| Bladder | 1 | 0 |
| Lung | 1 | 0 |
| Total | 59 | 98 |

**Figure 1** Percentage of low-grade patients of different ages remaining disease free after their initial treatment alone.

**Figure 2** Cause-specific survival of low-grade patients in different age groups.

under 60 years of age, only 43% are projected to have relapsed by 10 years, compared with 59% of those patients aged 60 years and over. The actuarial cause-specific survival at 10 years for the under-60s was 84% compared with 56% in those aged 60 years and over.

### High-grade disease

*Patient demographics* Two hundred and forty-three patients had high-grade disease, which was nodal in 145 cases (60%) and extranodal in 98 (40%): 45 patients had diffuse mixed-cell lymphoma and 198 patients diffuse large-cell/immunoblastic lymphoma. There were 124 males and 119 females. The median age was 56 years with a range of 17–84 years. The sites of disease are shown in Table I.

Twelve patients had a Hb level of $<12.0\ g\ dl^{-1}$ at presentation, 16 had an albumin of $<36\ g\ l^{-1}$ and 15 had an ESR of $>40\ mm\ h^{-1}$ or more. Seventy-four patients had a low lymphocyte count ($<1.5 \times 10^9/l^{-1}$) at presentation.

*Results of treatment* Two hundred and four patients (84%) achieved a CR maintained for at least 3 months after completion of radiotherapy. For the 39 patients not achieving a CR the cause was disease progression within the irradiated field in seven patients and disease appearing outside of the irradiation field in 32 patients. Of the patients achieving CR the actuarial relapse rate at 10 years was 32%, with all of the relapses occurring within 5 years. The resulting overall actuarial percentage of patients achieving CR and remaining disease free thereafter was 45% at 10 years.

Of the 80 patients who received second-line treatment (32 after induction failure and 48 after relapse), 33 achieved a second CR from it (41%) (after RT in 16 and after chemotherapy in 64).

The overall actuarial survival at 10 years was 61%, rising to 67% when patients dying from causes other than lymphoma or its treatment were excluded.

*Subgroup analysis* Multivariate analysis of those patients with high-grade disease who achieved complete remission from their initial treatment identified site of disease (nodal/extranodal) as the only significant factor related to relapse rate ($P<0.02$), patients with nodal involvement having a relatively high relapse rate. The CR rates of the different age groups were similar, with the exception of older patients [87%, 86%, 92% and 65% ($n=43$) for ages <50, 50–59, 60–69 and 70+ respectively]. The percentage of patients of different ages remaining disease free from their initial treatment alone is shown in Figure 3.

Multivariate analysis revealed age to be the only significant factor related to overall survival ($P<0.0001$): the older the

**Figure 3** Percentage of patients of different ages with high-grade disease remaining disease free after their initial treatment alone.

age the poorer the survival. The survival of patients in the different age groups is shown in Figure 4. Of patients under 60 years of age only 26% are projected to have relapsed by 10 years, compared with 42% of those patients aged 60 years and over. The actuarial cause-specific survival at 10 years was 80% for the under-60s and 52% for those aged 60 years and above.

*Comparison of low- and high-grade stage 1 disease* Patients with low- and high-grade lymphomas had a similar age and sex distribution. Inguinal disease was more common in patients with low-grade disease (32% *vs* 17%) ($P<0.00001$). Extranodal disease was more common in patients with high-grade disease (40% *vs* 28%) ($P<0.00001$). The sites of extranodal disease also tended to vary between the different histological grades, the tonsil and skin being the commonest sites of high-grade disease and the parotid and eye/orbit being the commonest sites of low-grade disease (Table I).

The complete response rate to radiotherapy was significantly higher in patients with low-grade than with high-grade disease ($P<0.0001$). There was no significant difference in relapse rate between the two grades ($P>0.2$). The overall cause-specific survival was significantly higher ($P<0.04$) in patients with low-grade disease than in those with high-grade disease, but this difference was mainly confined to the first 4 years after start of treatment, and there was no such difference between low- and high-grade disease in those patients who achieved CR ($P>0.9$).

The effects of age upon the relapse rate and the cause-specific survival in low- and high-grade disease are summarised in Table II.

### Analysis of the series as a whole

*Results of treatment* A total of 407 patients (90%) achieved complete remission from their initial treatment. Of these patients, 43% had relapsed within 10 years of the start of initial treatment. The resulting overall actuarial percentage of patients remaining clinically free from NHL (and therefore

**Figure 4** Cause-specific survival of patients with high-grade disease for different age groups.

**Table II** Effect of age on relapse rate and cause-specific survival at 10 years

| | *Low grade* | | *High grade* | |
|---|---|---|---|---|
| *Age (years)* | *Actuarial relapse rate (%)* | *Actuarial cause-specific survival (%)* | *Actuarial relapse rate (%)* | *Actuarial cause-specific survival (%)* |
| <50 | 38 | 90 | 28 | 81 |
| 50–59 | 49 | 77 | 25 | 79 |
| 60–69 | 47 | 65 | 39 | 57 |
| >70 | >84 | <34 | 49 | 31 |

probably permanently cured of their disease solely from initial treatment) was 52% at 10 years. The overall survival was 62%, and the overall cause-specific survival from NHL was 70% at 10 years.

Multivariate analysis identified age ($P<0.007$), site (nodal/extranodal, $P<0.003$), extranodal site ($P<0.02$) and nodal site ($P<0.05$) as significant factors related to relapse rate, with younger age, extranodal involvement, extranodal involvement of sites with high frequency of occurrence and nodal involvement of inguinal sites being related to relatively good prognosis. The percentage of patients of different ages remaining disease free after their initial treatment alone is shown in Figure 5.

For overall survival, multivariate analysis of the censored data revealed age ($P<0.0001$) as a significant factor related to survival, with increasing age being related to decreasing survival (Figure 6). In addition to age, multivariate analysis also identified histopathological grade as a significant prognostic factor ($P<0.01$). Of patients less than 60 years of age only 34% are projected to have relapsed by 10 years, compared with 53% of those patients aged 60 years and over. The actuarial cause-specific survival at 10 years for the under-60s was 82% (90% confidence interval 76–86%) compared with 54% (90% confidence interval 44–64) in patients of 60 years and over.

A small proportion of patients in the series had relatively low haemoglobin levels or relatively high ESRs at presentation. On univariate analysis these patients had a significantly

**Figure 5** Percentage of all patients of different ages remaining disease free after their initial treatment alone.

**Figure 6** Cause-specific survival of all patients for different age groups.

low overall survival compared with patients with normal levels, though the numbers of patients involved were considered to be too small to include in a multivariate analysis.

### Discussion

The overall survivals of 72% at 5 years and 62% at 10 years in the present series compare favourably with those for stage 1 patients of 76% at 10 years reported by Hagberg *et al.* (1989), 76% ('favourable' histopathology) at 10 years by Parayani *et al.* (1983) and 64% at 10 years by Timothy *et al.* (1980).

The major prognostic factor determining survival in this series of patients was the age of the patients at the start of their initial treatment. The percentage of patients over the age of 70 years remaining disease free after their initial treatment alone was low in both histopathological grades, and the cause-specific survival of these patients was less than 40% at 10 years for the series as a whole. This was because of a relatively high relapse rate (and a relatively low CR rate in high-grade disease), together with a relatively low success rate of salvage therapy. The overall cause-specific survival from NHL of patients under the age of 50 years was high, being of the order of 85% at 10 years for the series as a whole, while that of patients aged 50–59 was relatively high at over 75%, and that of patients aged 60–69 was lower at approximately 65%. For patients under 60 years the actuarial cause-specific survival was 82%, compared with 54% for patients of 60 years and over. The relatively high survival of younger patients was due to the combination of a relatively high cure rate from initial treatment with radiotherapy, together with a relatively high salvage rate of those patients with disease persisting after initial treatment. Age has been widely reported by other workers to be a significant prognostic factor in stage 1 patients and stage 1 and 2 patients combined who have all or mostly received initial treatment with RT. Thus Timothy *et al.* (1980) and Jeffrey *et al.* (1991) found age to be a significant prognostic factor in stage 1 patients on univariate analysis, as did Parayani *et al.* (1983), Sutcliffe *et al.* (1985), Kaminski *et al.* (1986) and Richards *et al.* (1989) in stage 1 and 2 patients on multivariate analysis.

The only other significant factor found on multivariate analysis to determine overall survival from NHL was histopathological grade, as has been reported after multivariate analysis by other workers (Parayani *et al.*, 1983; Kaminski *et al.*, 1986). The effect of this factor upon survival was mainly confined to the first few years after treatment, during which time the rate of attrition was greater in high-grade than in low-grade patients. This was because a greater proportion of patients with high-grade disease failed to achieve CR from initial RT and because of the relatively low success rate of further therapy in salvaging these patients. In other words, the significance of grade derived from its delineation of a group of patients in whom the CR rate from initial treatment was relatively low. In fact, the cause-specific overall survival of high-grade patients aged less than 60 was high, since poor salvage rates were mainly confined to the other patients. It is worth noting that relapses occurred predominantly in the first 5 years in both low- and high-grade disease, indicating that nearly half the patients in both categories were cured by initial radiotherapy alone.

The initial CR rate for the series overall was high, at 90%. Examination of patients who failed to achieve complete remission showed that most of these patients manifested further disease in sites other than those clinically involved at presentation, suggesting that these patients were not in fact 'true' stage 1 patients, but had occult disease in another site or sites at presentation. Multivariate analysis revealed that the prognostic factors related to relapse in the series overall were the presence of extranodal involvement and the age of the patient, and to a lesser extent the site of the extranodal and nodal involvement. Although extranodal involvement was not found to be a prognostic factor for overall survival, the cause-specific survival curve for patients with extranodal involvement plateaued out, in contrast to the survival curve for patients with nodal involvement, which showed a continuing rate of attrition. This suggests that the possibility of permanent cure is higher for patients with extranodal disease than for those with nodal disease. The significance of the relatively low relapse rate of patients with involvement of lymph nodes in the groin, and of patients with extranodal involvement of 'high frequency' sites, is unclear.

Overall, the long-term results in younger patients with stage 1 NHL treated initially with radiotherapy are relatively good in both low- and high-grade disease, the 10 year cause-specific survival exceeding 80% in both categories. It must be noted that the size of the nodal mass was not recorded in this series, and it may be that results are less satisfactory in those with bulky disease; in such patients we believe that combined chemotherapy and radiotherapy is advisable. The major question is whether initial chemotheapy or combined modality therapy would have been more effective than initial RT alone in these younger patients. The lack of evidence that chemotherapy can be curative in patients with more advanced low-grade disease does not encourage early chemotherapy in this situation (Nissen *et al.*, 1983). However, excellent results have been reported with initial chemotherapy in high-grade stage 1/1E disease. Jones *et al.* (1989) reported on the combined series from Tucson and Vancouver of 61 stage 1/1E patients treated with CHOP chemotherapy with or without involved-field chemotherapy. Almost all patients attained a complete remission, and with a median follow-up of just over 4 years there had been seven relapses. The actuarial overall survival at 5 years was approximately 90%. Longo *et al.* (1989) have reported on the results of Pro-MACE-MOPP followed by involved-field radiation therapy in 47 clinical stage 1/1E patients with high-grade lymphomas. Forty-five achieved a CR and with a median follow-up of 42 months none had relapsed. These results are most encouraging, but the overall survival results reported in the present paper for radiotherapy alone in the under-60s provide a challenging yardstick with which to judge the results of initial chemotherapy. We believe that the benefits of initial chemotherapy in younger patients overall remain unproven, although in future it may be possible with immunological or molecular markers to identify a subgroup of these patients who benefit from chemotherapy.

The long-term outcome of patients over 60 years of age with both low-grade and high-grade stage 1 NHLs was far less satisfactory than the younger patients and those over 70 years did particularly badly. Unfortunately, this group of patients, to whom one might most wish to administer chemotherapy, are least able to tolerate it. Advanced age itself has been shown to independently predict for a poor response to chemotherapy (Shipp *et al.*, 1992), although this was not found to be the case in stage 1 patients treated with CHOP (Jones *et al.*, 1989). Short-course treatments specifically designed for the elderly have been developed (O'Reilly *et al.*, 1990) and they merit exploration in clinical stage 1 high-grade NHL.

The BNLI would like to thank the collaborators from the referring centres whose patients are included in this analysis. We are grateful for financial help from the Lymphoma Research Trust, The Cancer Research Campaign, The Lisa Lear Fund, The Isle of Man Anti-Cancer Association, and to Miss S.P. Ray for typing the manuscript.

### References

COX, D.R. (1972). Regression models and life tables. *J. R. Stat. Soc. B*, **34**, 187–220.

CRELLIN, A.M., VAUGHAN HUDSON, B., BENNETT, M.H., HARLAND, S. & VAUGHAN HUDSON, G. (1993). Non-Hodgkin's lymphoma of the testis. *Radiother. Oncol.*, **27**, 99–106.

HAGBERG, H., PETTERSON, U., GLIMELIUS, B. & SANDSTROM, C. (1989). Prognostic factors in Non-Hodgkin's lymphoma stage 1 treated with radiotherapy. *Acta Oncol.*, **28**, Fasc. 1, 45–50.

HALLAHAN, D.E., FARAH, H., VOKES, E.E., BITRAN, J.D., ULTMANN, J.E., GOLOMB, H.M. WEICHSELBAUM, R.R. (1989). The problems of failure in patients with pathological stage I and II diffuse histocytic lymphoma treated with radiation therapy alone. *Int. J. Rad. Oncol. Biol. Phys.*, **17**, 767–771.

ISAACSON, P.G. & WRIGHT, D.H. (1984). Extranodal malignant lymphoma arising from mucosa-associated lymphoid tissue. *Cancer*, **53**, 2515–2524.

ISAACSON, P.G. & SPENDER, J. (1988). Malignant lymphoma of mucosa-associated lymphoid tissue. In *Malignant Lymphomas*, Habeshaw, J.A. & Lauder, I. (eds). pp. 170–200. Churchill Livingstone: Edinburgh.

JEFFREY, G.M., MEAD, G.M., WHITEHOUSE, J.M.A. & RYALL, R.D.H. (1991). Involved field radiotherapy or chemotherapy in the management of stage I nodal intermediate grade non-Hodgkin's lymphoma. *Br. J. Cancer*, **64**, 933–937.

JONES, S.E., MILLER, T.P. & CONNORS, J.M. (1989). Long-term follow-up and analysis for prognostic factors for patients with limited-stage diffuse large-cell lymphoma treated with initial chemotherapy with or without adjuvant radiotherapy. *J. Clin. Oncol.*, **7**, 1186–1191.

KAMINSKI, M.S., COLEMAN, C.N., COLBY, T.V., COX, R.S. & ROSENBERG, S.A. (1986). Factors predicting survival in adults with stage I and II large-cell lymphoma treated with primary radiation therapy. *Ann. Intern. Med.*, **104**, 747–756.

LONGO, D.L., GLATSTEIN, E., DUFFEY, P.L., IHDE, D.C., HUBBARD, S.M., FISHER, R.I., JAFFE, E.S., GILLIOM, M., YOUNG, R.C. & DE VITA Jr, V.T. (1989). Treatment of localised aggressive lymphoma with combination chemotherapy followed by involved-field radiation therapy. *J. Clin. Oncol.*, **7**, 1295–1302.

MILLER, T.D. & JONES, S.E. (1980). Is there a role for radiotherapy in localised diffuse lymphomas? *Cancer Chemother. Pharmacol.*, **4**, 67–70.

MORTON, J.E., LEYLAND, M.J., VAUGHAN HUDSON, G., VAUGHAN HUDSON, B., ANDERSON, L., BENNETT, M.H. & MACLENNAN, K.A. (1993). Primary gastrointestinal non-Hodgkin's lymphoma: a review of 175 British National Lymphoma Investigation cases. *Br. J. Cancer*, **67**, 776–782.

NISSEN, N.I. & ERSBOLL, J. (1985). Treatment of Non-Hodgkin's lymphoma in adults. In *Leukaemia and Lymphomas*. Wiernik, P.H. (ed.). pp. 97–126. Churchill Livingstone: New York.

NISSEN, N.I., ERSBOLL, J., HANSEN, H.S., WALBOHM-JORGENSEN, S., PEDERSEN-BJERGAARD, J., HANSEN, M.M. & RYGARD, J. (1983). A randomised study of radiotherapy versus radiotherapy plus chemotherapy in stage 1–2 Non-Hodgkin's lymphoma. *Cancer*, **52**, 1–7.

O'REILLY, S.E. & CONNORS, J.M. (1992). Non-Hodgkin's lymphoma. I: characterisation and treatment. *BMJ*, **304**, 1682–1686.

O'REILLY, S.E., HASKINS, P., KLASA, R., KLIMO, P. & CONNORS, J.M. (1990). Chemotherapy for elderly patients with advanced stage large cell lymphoma – a little goes a long way (abstracts). 4th International Conference on Malignant Lymphoma, Lugano. No. 59.

PARYANI, S.B., HOPPE, R.T., COX, R.S., COLBY, T.V., ROSENBERG, S.A. & KAPLAN, H.S. (1983). Analysis of non-Hodgkin's lymphomas with nodular and favourable histologies, stages I and II. *Cancer*, **52**, 2300–2307.

PETO, R., PIKE, M.C., ARMITAGE, P., BRESLOW, N.E., COX, D.R., HOWARD, S.V., MANTEL, N., MCPHERSON, K., PETO, J. & SMITH, P.G. (1971). Design and analysis of randomised clinical trials requiring prolonged observations of each patient. II. Analysis and examples. *Br. J. Cancer*, **35**, 1–39.

RICHARDS, M.A., GREGORY, W.M., HALL, P.A., DHALIWAL, H.S., FERNANDEZ, J., STANSFIELD, A.G., JONES, A.E. & LISTER, T.A. (1989). Management of localised non-Hodgkin's lymphoma: the experience at St Bartholomew's Hospital 1972–1985. *Haematol. Oncol.*, **7**, 1–18.

SHIPP, M., HARRINGTON, D., CHAIRPERSONS - ANDERSON, J., ARMITAGE, J., BONADONNA, G., BRITTINGER, F., CABANILLAS, G., COIFFIER, B., CONNORS, J., COWAN, R., CROWTHER, D., ENGELHARD, M., FISHER, R., GISSELBRECHT, C., HORNING, S., LEPAGE, E., LISTER, A., MEERWALDT, J., MONTSERRAT, E., NISSEN, N., OKEN, M., PETERSON, B., TONDINI, C., VELASQUEZ, W. & YEAP, B. (1992). Development of a predictive model for aggressive lymphoma: the international NHL prognostic factors project. *Proc. ASCO*, **11**, 1084.

SUTCLIFFE, S.B., GOSPODAROWICZ, M.K., BUSH, R.S., BROWN, T.C., CHUA, T., BEAN, H.A., CLARK, R.M., DEMBO, A., FITZPATRICK, P.J. & PETERS, V. (1985). Role of radiation therapy in localised non-Hodgkin's lymphoma. *Radiother. Oncol.*, **4**, 211-223.

TIMOTHY, A.R., LISTER, T.A., KATZ, D. & JONES, A.E. (1980). Localised non-Hodgkin's lymphoma. *Eur. J. Cancer*, **16**, 799-807.

VOKES, E.E., ULTMAN, J.E., GOLOMB, H.M., GAYNOR, E.R., FERGUSON, D.J., GRIEM, M.L. & OLESKE, D. (1985). Long term survival of patients with localised diffuse histocytic lymphoma. *J. Clin. Oncol.*, **3**, 1309-1317.


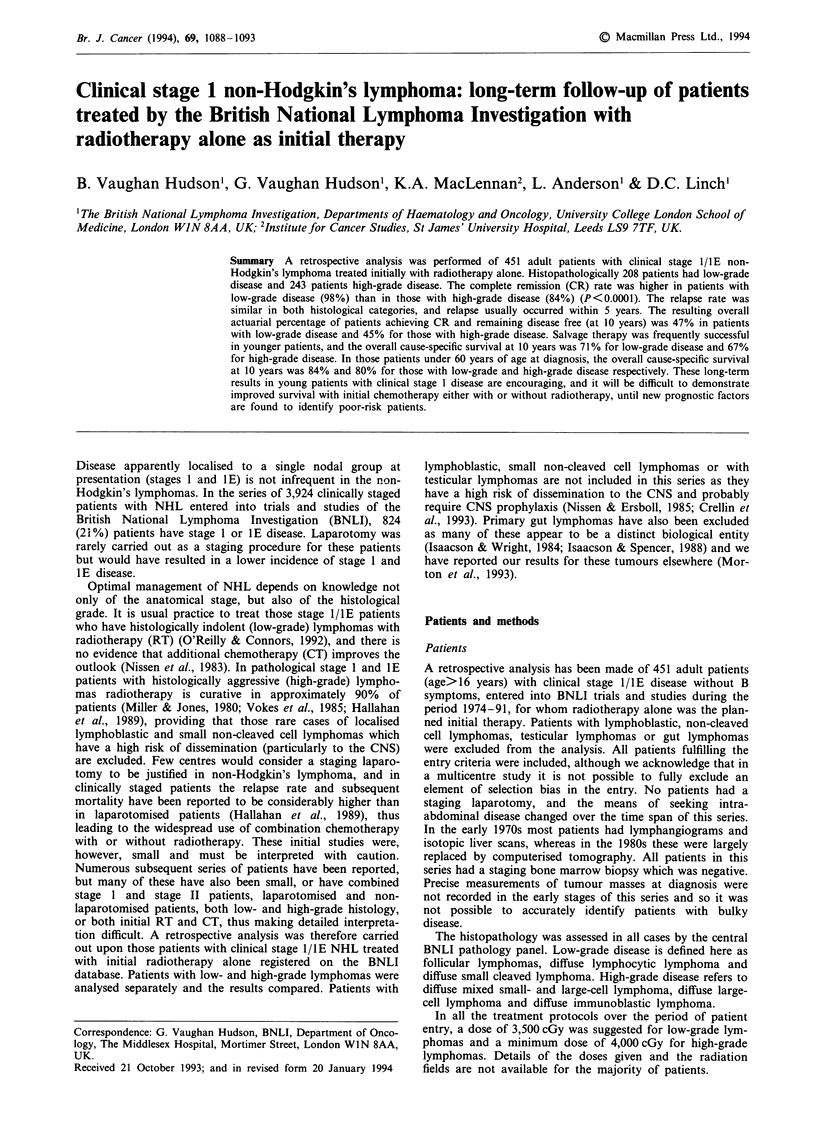

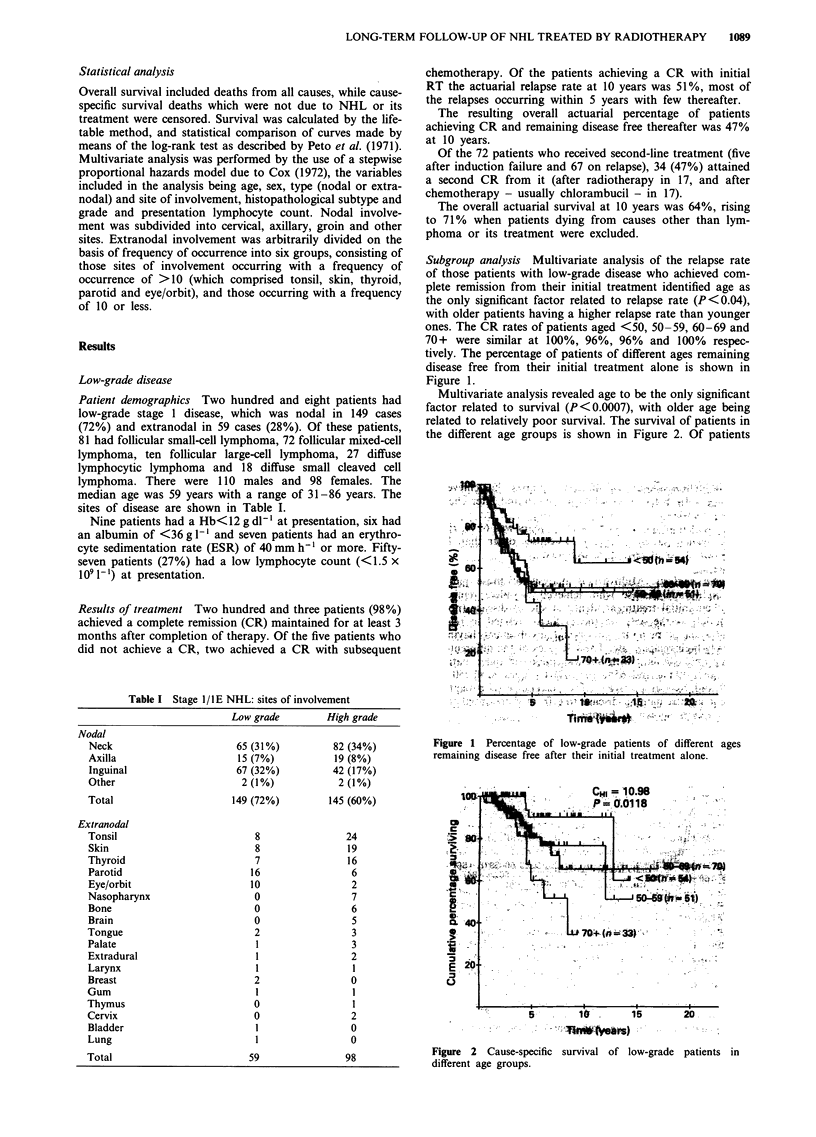

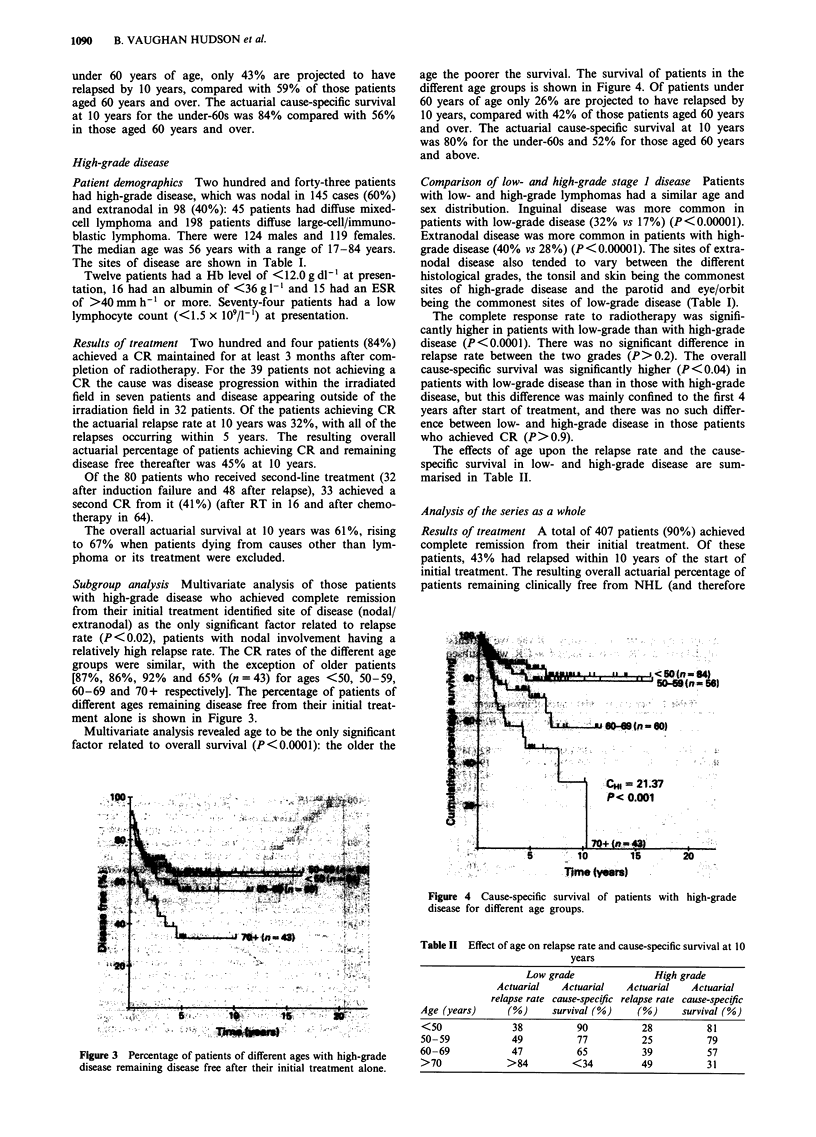

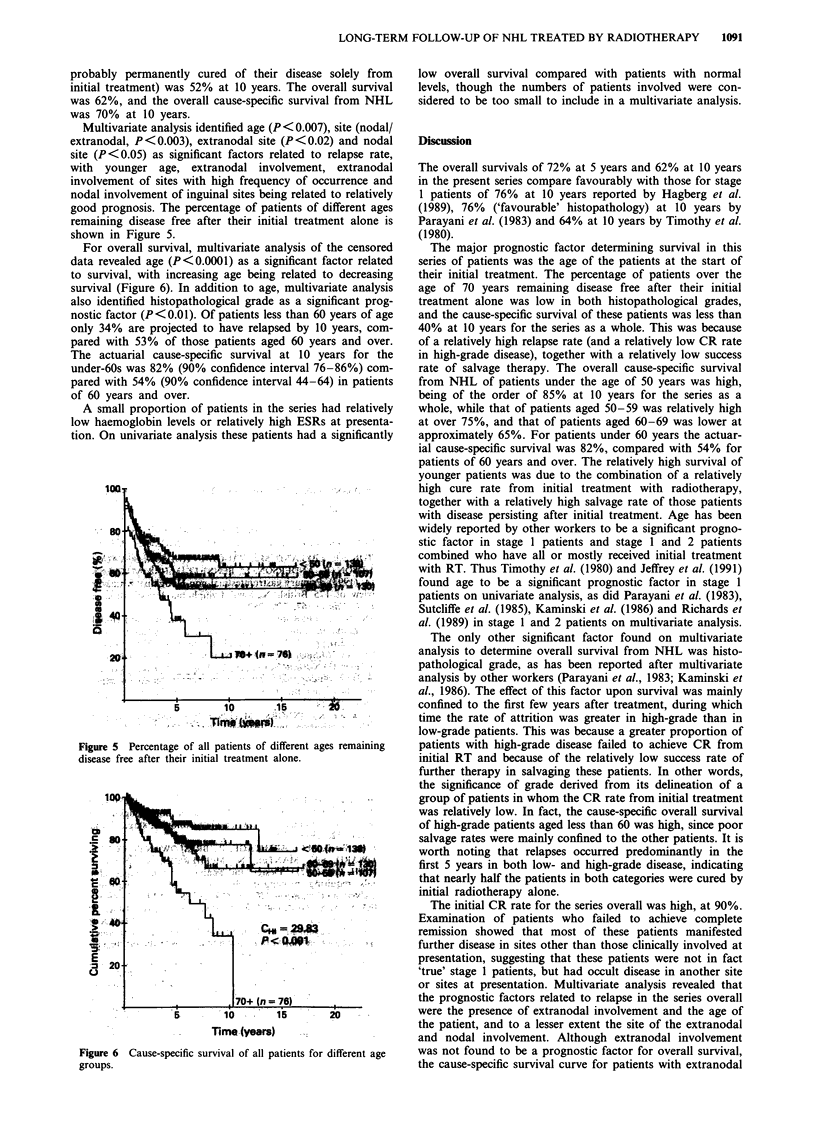

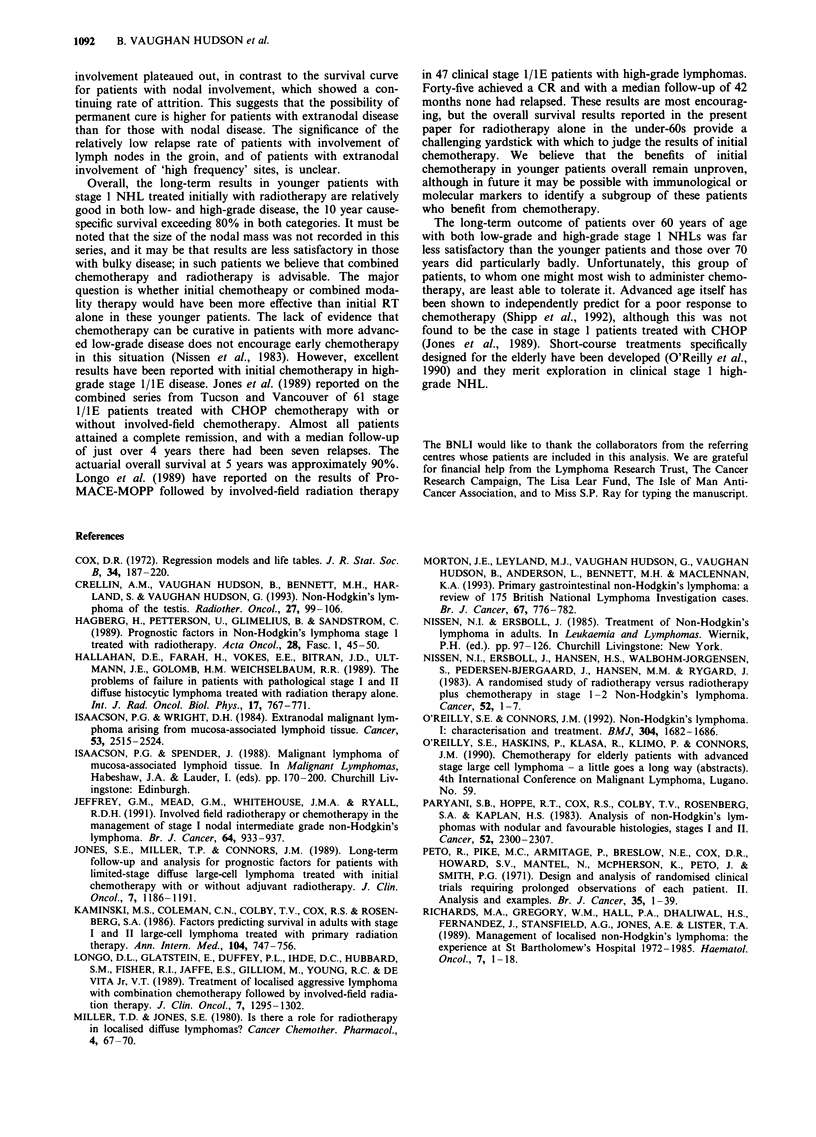

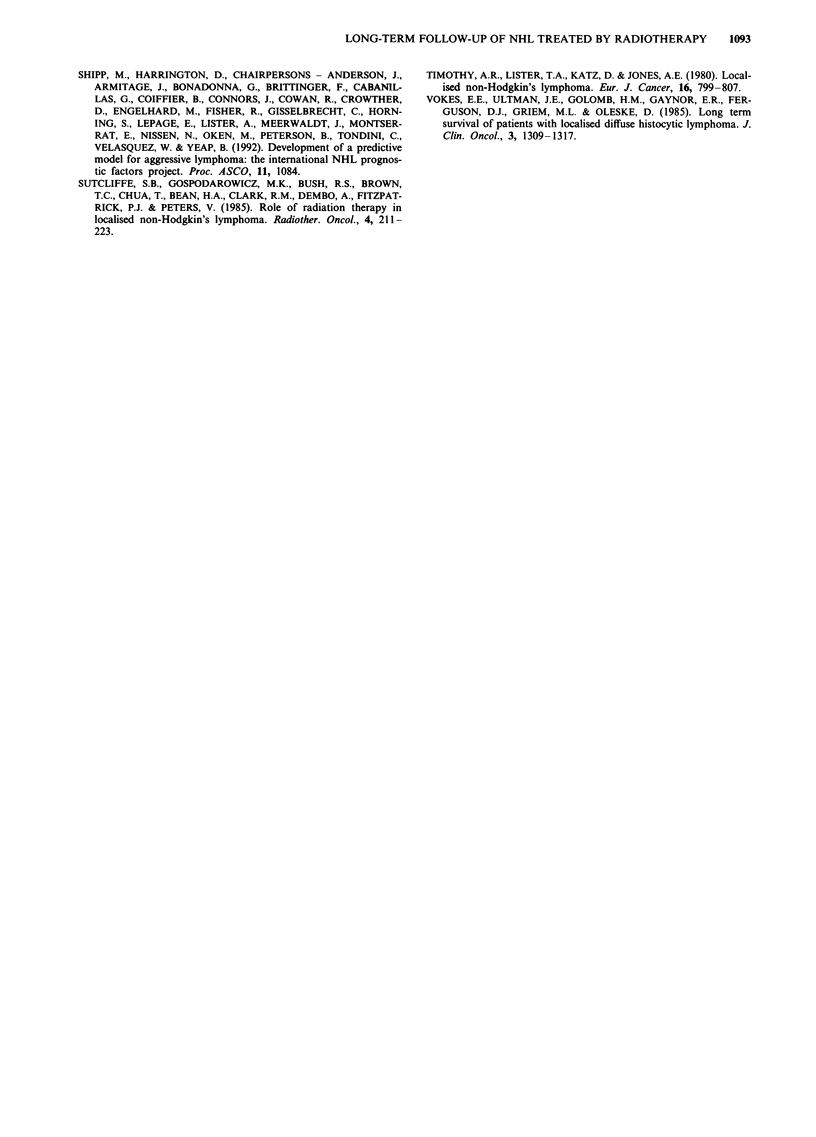


## References

[OCR_00722] Crellin A. M., Hudson B. V., Bennett M. H., Harland S., Hudson G. V. (1993). Non-Hodgkin's lymphoma of the testis.. Radiother Oncol.

[OCR_00725] Hagberg H., Pettersson U., Glimelius B., Sundström C. (1989). Prognostic factors in non-Hodgkin lymphoma stage I treated with radiotherapy.. Acta Oncol.

[OCR_00730] Hallahan D. E., Farah R., Vokes E. E., Bitran J. D., Ultmann J. E., Golomb H. M., Weichselbaum R. R. (1989). The patterns of failure in patients with pathological stage I and II diffuse histiocytic lymphoma treated with radiation therapy alone.. Int J Radiat Oncol Biol Phys.

[OCR_00737] Isaacson P., Wright D. H. (1984). Extranodal malignant lymphoma arising from mucosa-associated lymphoid tissue.. Cancer.

[OCR_00748] Jeffery G. M., Mead G. M., Whitehouse J. M., Ryall R. D. (1991). Involved field radiotherapy or chemotherapy in the management of stage I nodal intermediate grade non-Hodgkin's lymphoma.. Br J Cancer.

[OCR_00754] Jones S. E., Miller T. P., Connors J. M. (1989). Long-term follow-up and analysis for prognostic factors for patients with limited-stage diffuse large-cell lymphoma treated with initial chemotherapy with or without adjuvant radiotherapy.. J Clin Oncol.

[OCR_00763] Kaminski M. S., Coleman C. N., Colby T. V., Cox R. S., Rosenberg S. A. (1986). Factors predicting survival in adults with stage I and II large-cell lymphoma treated with primary radiation therapy.. Ann Intern Med.

[OCR_00767] Longo D. L., Glatstein E., Duffey P. L., Ihde D. C., Hubbard S. M., Fisher R. I., Jaffe E. S., Gilliom M., Young R. C., DeVita V. T. (1989). Treatment of localized aggressive lymphomas with combination chemotherapy followed by involved-field radiation therapy.. J Clin Oncol.

[OCR_00774] Miller T. P., Jones S. E. (1980). Is there a role for radiotherapy in localized diffuse lymphomas?. Cancer Chemother Pharmacol.

[OCR_00781] Morton J. E., Leyland M. J., Vaughan Hudson G., Vaughan Hudson B., Anderson L., Bennett M. H., MacLennan K. A. (1993). Primary gastrointestinal non-Hodgkin's lymphoma: a review of 175 British National Lymphoma Investigation cases.. Br J Cancer.

[OCR_00791] Nissen N. I., Ersbøll J., Hansen H. S., Walbom-Jørgensen S., Pedersen-Bjergaard J., Hansen M. M., Rygård J. (1983). A randomized study of radiotherapy versus radiotherapy plus chemotherapy in stage I-II non-Hodgkin's lymphomas.. Cancer.

[OCR_00798] O'Reilly S. E., Connors J. M. (1992). Non-Hodgkin's lymphoma. I: Characterisation and treatment.. BMJ.

[OCR_00809] Paryani S. B., Hoppe R. T., Cox R. S., Colby T. V., Rosenberg S. A., Kaplan H. S. (1983). Analysis of non-Hodgkin's lymphomas with nodular and favorable histologies, stages I and II.. Cancer.

[OCR_00815] Peto R., Pike M. C., Armitage P., Breslow N. E., Cox D. R., Howard S. V., Mantel N., McPherson K., Peto J., Smith P. G. (1977). Design and analysis of randomized clinical trials requiring prolonged observation of each patient. II. analysis and examples.. Br J Cancer.

[OCR_00822] Richards M. A., Gregory W. M., Hall P. A., Dhaliwal H. S., Fernandez J., Stansfeld A. G., Jones A. E., Lister T. A. (1989). Management of localized non-Hodgkin's lymphoma: the experience at St. Bartholomew's Hospital 1972-1985.. Hematol Oncol.

[OCR_00845] Sutcliffe S. B., Gospodarowicz M. K., Bush R. S., Brown T. C., Chua T., Bean H. A., Clark R. M., Dembo A., Fitzpatrick P. J., Peters M. V. (1985). Role of radiation therapy in localized non-Hodgkin's lymphoma.. Radiother Oncol.

[OCR_00849] Timothy A. R., Lister T. A., Katz D., Jones A. E. (1980). Localized non-Hodgkin's Lymphoma.. Eur J Cancer.

[OCR_00855] Vokes E. E., Ultmann J. E., Golomb H. M., Gaynor E. R., Ferguson D. J., Griem M. L., Oleske D. (1985). Long-term survival of patients with localized diffuse histiocytic lymphoma.. J Clin Oncol.

